# The accuracy of hospital ICD-9-CM codes for determining Sickle Cell Disease genotype

**Published:** 2017-07-28

**Authors:** Angela B. Snyder, Peter A. Lane, Mei Zhou, Susan T. Paulukonis, Mary M. Hulihan

**Affiliations:** 1 Georgia State University, Department of Public Management and Policy, Atlanta, GA and Georgia State University, Georgia Health Policy Center, Atlanta, GA, USA; 2 Aflac Cancer and Blood Disorders Center, Children's Healthcare of Atlanta, Atlanta, GA and Department of Pediatrics, Emory University School of Medicine, Atlanta, GA, USA; 3 Georgia State University, Georgia Health Policy Center, Atlanta, GA, USA; 4 Public Health Institute, Richmond, CA, USA; 5 Centers for Disease Control and Prevention, Division of Blood Disorders, Atlanta, GA, USA

**Keywords:** Sickle cell disease, Sickle cell anemia, Genotype, Administrative data, Surveillance, ICD-9-CM codes

## Abstract

Sickle cell disease affects more than 100,000 individuals in the United States, among whom disease severity varies considerably. One factor that influences disease severity is the sickle cell disease genotype. For this reason, clinical prevention and treatment guidelines tend to differentiate between genotypes. However, previous research suggests caution when using a claimsbased determination of sickle cell disease genotype in healthcare quality studies.

The objective of this study was to describe the extent of miscoding for the major sickle cell disease genotypes in hospital discharge data. Individuals with sickle cell disease were identified through newborn screening results or hemoglobinopathy specialty care centers, along with their sickle cell disease genotypes. These genotypes were compared to the diagnosis codes listed in hospital discharge data to assess the accuracy of the hospital codes in determining sickle cell disease genotype. Eighty-three percent (sickle cell anemia), 23% (Hemoglobin SC), and 31% (Hemoglobin Sβ^+^ thalassemia) of hospitalizations contained a diagnosis code that correctly reflected the individual's true sickle cell disease genotype. The accuracy of the sickle cell disease genotype coding was indeterminate in 11% (sickle cell anemia), 12% (Hemoglobin SC), and 7% (Hemoglobin Sβ^+^ thalassemia) and incorrect in 3% (sickle cell anemia), 61% (Hemoglobin SC), and 52% (Hemoglobin Sβ^+^ thalassemia) of the hospitalizations. The use of ICD-9-CM codes from hospital discharge data for determining specific sickle cell disease genotypes is problematic. Research based solely on these or other types of administrative data could lead to incorrect understanding of the disease.

## Background

Sickle cell disease (SCD) is characterized by chronic hemolytic anemia and a wide variety of acute and chronic complications caused by intermittent episodes of vaso-occlusion, vascular injury, and organ damage. SCD affects more than 100,000 individuals in the United States^1^ and with recent advances in care, individuals with SCD are living longer^2^. Even so, disease severity varies considerably among individuals. While some exhibit severe complications and die before reaching middle age, others are far less symptomatic^3^.

One factor that influences disease severity is the SCD genotype. In the United States, most individuals with SCD have homozygous hemoglobin (Hb) SS disease (Hb SS), and the remainder have a compound heterozygous form of SCD caused by co-inheritance of Hb S with a different beta globin mutation such as Hb C or a variety of β thalassemia mutations^4^. The Hb Sβ thalassemias are divided into two groups depending on the severity of the β thalassemia mutation; mutations that result in absent production of β globin are termed Hb Sβ^0^ thalassemia while those that result in reduced, but not absent, production of β globin are termed Hb Sβ^+^ thalassemia. Individuals with Hb SS or Hb Sβ^0^ thalassemia are classified as having sickle cell anemia (SCA) because their hematological phenotype is characterized by a more severe chronic hemolytic anemia than those with Hb SC or Hb Sβ^+^ thalassemia, who have less hemolysis and less severe or no anemia. In the United States, Hb SC is the most prevalent of the compound heterozygous SCD genotypes, followed by Hb Sβ^+^ thalassemia and then Hb Sβ^0^ thalassemia. A recent population-based surveillance study from six states found that 61.4% of individuals with SCD had SCA, 28.3% had Hb SC, 8.5% had Hb Sβ^+^ thalassemia, and 1.8% had another compound heterozygous form of SCD^5^.

It is sometimes difficult to distinguish between the two SCA genotypes because the co-inheritance of α thalassemia in persons with Hb SS results in significant overlap of complete blood count and hemoglobin electrophoresis results in persons with Hb Sβ^0^ thalassemia. Therefore, the genotypes cannot be reliably differentiated without DNA analysis. However, the distinction between SCA and Hb SC/Hb Sβ^+^ thalassemia is important because the risk for some of the more severe complications of SCD, such as stroke, is much higher in SCA than in Hb SC/Hb Sβ^+^ thalassemia, especially during childhood^6^. For this reason, clinical prevention and treatment guidelines tend to differentiate between genotypes in their practice recommendations based on evidence of clinical effectiveness and differing risk- benefit trade-offs. For example, annual transcranial Doppler screening for prevention of stroke in childhood is recommended for children with SCA but not for those with Hb SC or Hb Sβ^+^ thalassemia. Similarly, indications for use of hydroxyurea are much broader and more inclusive for SCA compared to Hb SC or Hb Sβ^+^ thalassemia^7^.

International Classification of Diseases, Ninth Revision, Clinical Modification (ICD-9-CM) codes have been commonly used by public health officials and health services researchers to identify individuals with SCD to evaluate complications, adherence to treatment guidelines, and the quality of care received. Because researchers have questioned the accuracy of SCD genotypes based on ICD-9-CM codes^8,9^, studies usually identify patients based on any SCD code (282.41-42, 282.6, 282.60-64, 282.68-69) in any position, primary, secondary or other^10-13^. A few of these studies have reported results by genotype, including reports detailing rehospitalizations^14^, influenza^15^, stroke^16^, and surgical outcomes for individuals with SCD^17^. Furthermore, some studies have been restricted to individuals with Hb SS codes only^18-20^ and others were limited to only individuals with ICD-9-CM codes for SCD crisis^21-24^. In only one instance has the accuracy of coding by SCD genotype been reported^25^. In that report, findings from an analysis of emergency department visits at a children's hospital found that ICD-9-CM codes for Hb SS were 87% accurate, but the Hb SS codes were very often used for ED visits of patients with other genotypes. Therefore, the authors suggested caution when using a claims-based determination of SCD genotype in healthcare quality studies.

Georgia and California have a unique set of SCD surveillance data collected through the Registry and Surveillance System for Hemoglobinopathies (RuSH)^5^. These population-based data can be used to test the accuracy of hospital discharge data for identifying SCD genotypes and, in turn, their utility in evaluating adherence to practice recommendations that differ by genotype. This is possible by comparing ICD-9-CM codes from the hospital discharge data to laboratory-based information collected from clinical sites and state newborn screening programs within the RuSH surveillance datasets. The purpose of this study was to use the hospital discharge records of a population-based sample to (1) describe the extent of miscoding for the major SCD genotypes and estimate the accuracy of ICD-9-CM codes and (2) report the positive predictive value of using Hb SS ICD-9-CM codes only (282.61 and 282.62) to identify individuals with SCA.

## Methods

The methods that California and Georgia used to collect and link population-based SCD surveillance data for 2004 to 2008 from a variety of sources were previously described^5^. The analyses presented here include individuals with SCD who were reported by newborn screening and/or one of the participating hemoglobinopathy specialty treatment centers (Georgia: Georgia Comprehensive Sickle Cell Center at Grady Memorial, Georgia Health Sciences University, and all three campuses of Children's Healthcare of Atlanta; California: University of California (UC) San Francisco Benioff Children's Hospital Oakland and San Francisco, Children's Hospital Los Angeles, UC Irvine Medical Center, Rady UC San Diego, UC Davis Medical Center) with a laboratory-confirmed diagnosis based on results of clinical laboratory evaluation that included quantitative hemoglobin identification by hemoglobin electrophoresis, high-performance liquid chromatography or DNA analysis. These individuals were then linked to inpatient files from each state's hospital discharge data (all records from 2004 to 2008) using multiple patient identifiers. This study only included individuals who had at least one hospitalization reported during the five year period.

**Figure 1:**
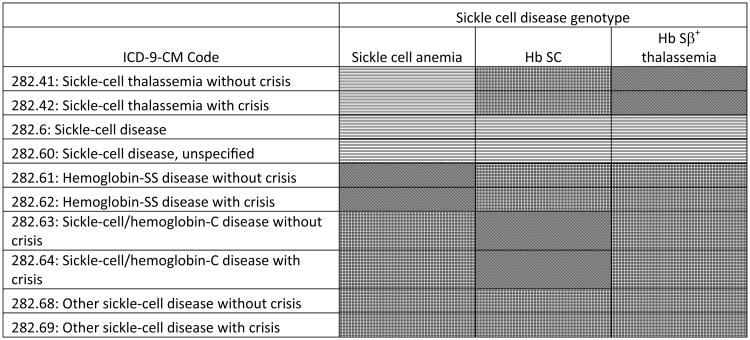
Comparison of known sickle cell disease (SCD) genotype and SCD ICD-9-CM hospital discharge code, California and Georgia, 2004-2008. Correct = dots; indeterminate = horizontal lines; incorrect = crosshatch

The hospital discharge data from Georgia included a maximum of ten diagnosis codes per admission; in the California data there were up to 25 diagnosis codes per admission. All of the diagnosis codes were scanned for the presence of an SCD ICD-9-CM code. These SCD codes were then compared to the known SCD genotype for the individual, and a determination of “correct,” “indeterminate,” “incorrect,” or “no code” was made, based on whether or not the diagnosis code and the known SCD genotype were considered to be a correct match (Figure 1). These categorizations were defined from the perspective of the known SCD genotype. That is, for a hospitalization of an individual with SCA, the “sickle-cell thalassemia with/without crisis” (282.41, 282.42) ICD-9-CM codes would be “correct” if the individual had Hb Sβ^0^ thalassemia, but incorrect if the individual had Hb SS, so the comparison was termed “indeterminate.” On the other hand, a hospitalization of an individual with Hb Sβ^+^ thalassemia would be termed “correct,” if the same codes were listed.

Hospital admissions for individuals with SCD genotypes other than Hb SS/Hb Sβ^0^ thalassemia, Hb SC, or Hb Sβ^+^ thalassemia are provided in Table 1, but were excluded from further analyses (n=78 California; n=68 Georgia). Admissions that included more than one SCD related ICD-9-CM diagnosis code were also removed (n=18 California, n=119 Georgia). The data analysis for this study was generated using SAS® version 9.3 (SAS Institute, 2010).

## Results

The RuSH surveillance project identified a total of 6,264 individuals with a known SCD genotype in California and Georgia; 1,976 in California and 4,288 in Georgia. Of these, 3,961 (63.2%) accounted for a total of 27,439 hospitalizations during 2004 through 2008 (Table 1); 43.2% were for pediatric patients aged 18 years or younger at the time of admission, and 46.8% were male patients. Individuals with SCA accounted for 72.5% of the patients with a known genotype and 79.9% of the hospital discharges; Hb SC accounted for 21.4% of patients and 15.3% of discharges; Hb Sβ^+^ thalassemia accounted for 5.5% of patients and 4.2% of discharges.

**Figure 2:**
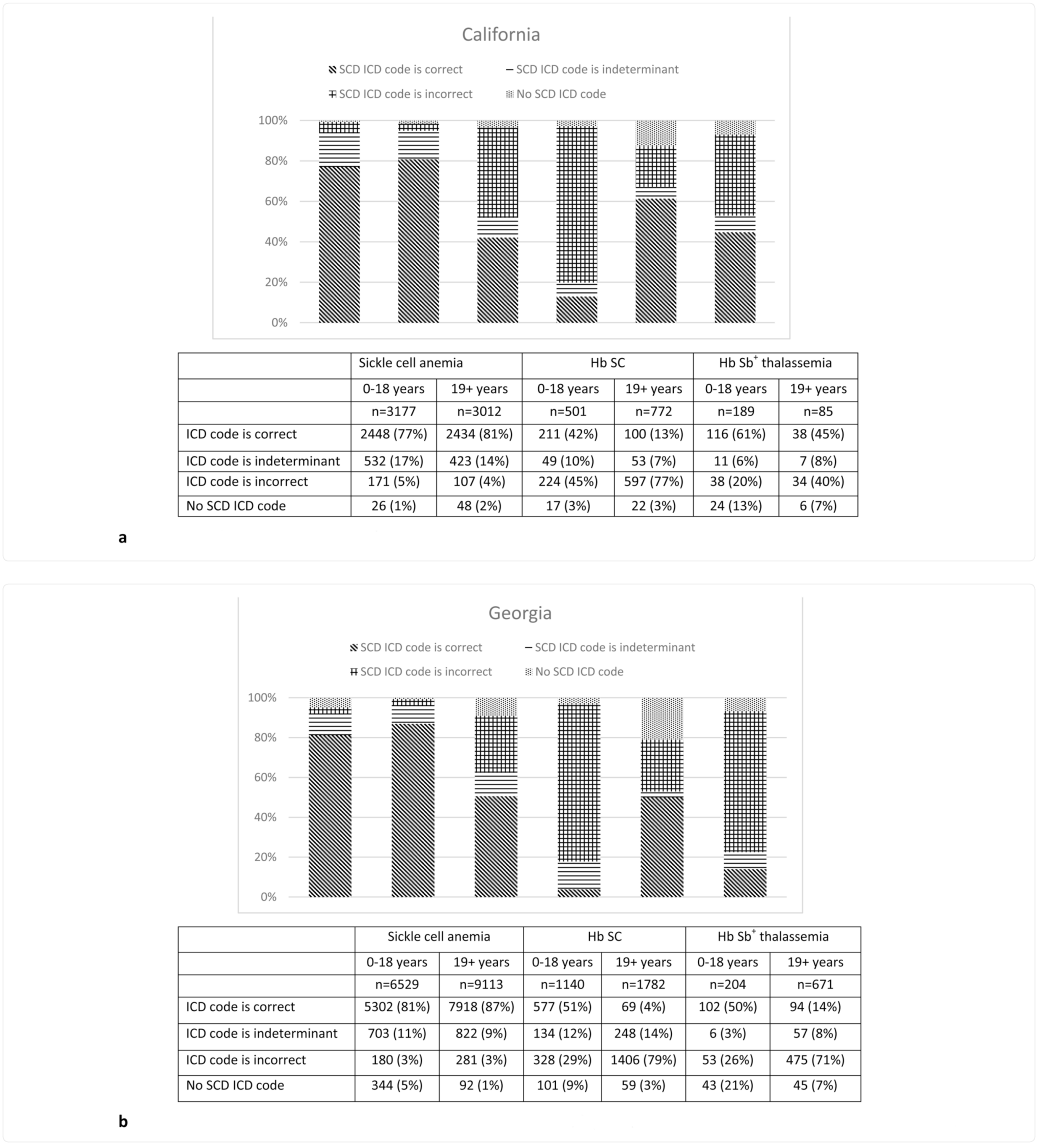
**a**. Accuracy of ICD-9-CM hospital discharge codes for determining sickle cell disease genotype, California, 2004-2008. **b**. Accuracy of ICD-9-CM hospital discharge codes for determining sickle cell disease genotype, Georgia, 2004-2008.

ICD-9-CM coding correctly identified SCA genotypes in 82.9% of hospitalizations (78.9% in CA, 84.5% in GA), while the coding was incorrect in 3.4% (4.5% in CA, 2.9% in GA) and indeterminate in 11.4% (15.4% in CA, 9.7% in GA). However, coding for Hb SC was correct in only 22.8% (24.4% in CA, 22.1% in GA) of hospitalizations, incorrect in 60.9% (64.5% in CA, 59.3% in GA) and indeterminate in 11.5% (8.0% CA, 13.1% GA). Individuals with Hb Sβ^+^ thalassemia were correctly coded in only 30.5% of hospitalizations overall; substantially higher in CA (56.2%) than in GA (22.4%). Coding for Hb Sβ^+^ thalassemia was incorrect in 52.2% (26.3% in CA, 60.3% in GA), and indeterminate in 7.0% (6.6% in CA, 7.2% in GA) (Figure 2). Two percent of hospitalizations for individuals with SCD in California and 4% in Georgia did not contain an SCD ICD-9-CM diagnosis code. The lack of any SCD diagnosis code was higher for Hb Sβ^+^ thalassemia (10.3%) compared with Hb SC (4.7%) and SCA (2.3%).

In our cohort of hospitalizations for individuals with SCD from both California and Georgia, using Hb SS ICD-9-CM codes only (282.61, 282.62) versus any SCD code to identify a pediatric patient with SCA improved the positive predictive value (PPV) from 84% (9,850/11,740) to 93% (7,750/8,306); however, in the adult population the PPV only slightly improved from 79% (12,229/15,435) to 81% (10,352/12,752) (Table 2).

## Discussion

One of the most important distinctions to make, when treating patients with SCD or evaluating their health and healthcare utilization patterns, is between patients with SCA genotypes and patients with other forms of SCD, because patients with SCA are at a higher risk for many disease-related complications and hospitalization^14,26^. However, the use of ICD-9-CM codes for determining specific SCD genotypes is problematic for multiple reasons. First, although it is sometimes difficult to distinguish Hb SS from Hb Sβ^0^ thalassemia based on results of hemoglobin electrophoresis or other non DNA tests, there is no single ICD-9-CM code for SCA, rather there are separate codes for “hemoglobin-SS disease with/without crisis” (282.61, 282.62) and “sickle-cell thalassemia with/without crisis” (282.41, 282.42). Furthermore, the latter ICD-9-CM codes do not differentiate between Hb Sβ^0^ thalassemia and Hb Sβ^+^ thalassemia, the former genotype causing SCA, while the latter does not. The difficulty in distinguishing between SCA genotypes, even with the presence of laboratory-based results, is the reason that the SCD surveillance data collected by Georgia and California, and used for the analyses presented here, utilized an SCA classification, rather than separate Hb SS and Hb Sβ^0^ thalassemia genotypes. Moreover, the limitations of the ICD-9-CM codes for determining SCD genotypes were the reason that the SCD surveillance data collected on individuals without a newborn screening report or diagnosis from a hemoglobinopathy specialty treatment center did not contain genotype information and were, therefore, excluded from this analysis.

In addition to the inherent limitations of the SCD ICD-9-CM codes themselves, our study indicates that there is also misuse of these codes, as has been previously reported^25^. That study from a single pediatric emergency department noted that the accuracy of correctly identifying individuals with Hb SS was 87%, with sensitivity, specificity, and PPV of 87%, 79%, and 87% respectively. Our study showed the accuracy of identifying individuals with SCA in hospital discharge data was similar. However the accuracy of ICD-9-CM codes to identify other SCD genotypes was poor, especially for individuals with Hb SC; over 75% of their hospitalizations contained an ICD-9-CM code that did not match their true SCD genotype. For Hb Sβ^+^ thalassemia, accuracy was also low (69%). This miscoding may be, in part, because some healthcare providers and coders do not appreciate the differences among SCD genotypes, leading to the use of Hb SS codes for non-Hb SS genotypes. While these analyses were based on ICD-9-CM codes, it is important to recognize that the ICD-10-CM codes for SCD that are now being used have similar limitations.

The lack of SCA ICD-9-CM codes, ambiguous codes for Hb Sβ^0^ thalassemia vs Hb Sβ^+^ thalassemia, and the high rates of mismatch between true SCD genotype and the SCD ICD-9-CM codes in hospital discharge data may lead to the erroneous interpretation of such data and mistaken conclusions. Using hospital discharge data to analyze hospital admission and/or readmission rates, the rate of a particular surgical procedure, or adherence to recommended therapy by genotype could incorrectly classify up to 15% of individuals with SCA, three-fourths of those with Hb SC, and over two-thirds of those with Hb Sβ^+^ thalassemia. Errors such as these could lead to incorrect understanding of the disease and adherence to recommended management and treatment. Our results suggest that caution should be exercised when interpreting research that relies solely on hospital discharge data to identify and determine the genotype of individuals with SCD.

The results presented here further suggest that using only Hb SS ICD-9-CM codes to identify individuals with SCA improves the genotypic accuracy of the resulting sample for pediatric patients when compared to using all SCD ICD-9-CM codes, but does little to improve identification of adults with SCA. These results are due, in part, to the high rates of miscoding for non-SCA genotypes, as well as the higher rates of hospitalization in individuals with SCA (80% of the SCD hospitalizations in this analysis, but only 73% of the patients with a hospitalization and 64% of all identified individuals with SCD, regardless of hospitalization).

A fundamental strength of this study is the identification of cohort members through population-based surveillance, rather than identification solely in administrative data or a SCD specialty clinic(s). While the use of administrative data alone requires fewer resources, it is often incomplete and may suffer from suboptimal quality. Data from SCD specialty clinics have higher quality and more complete reporting, but contain a limited number of cases and may not represent a population-based sample of all patients.

This analysis also has limitations. It has a narrow focus on hospital discharge data, thus excluding the 37.7% of patients with known SCD genotypes who were not hospitalized during the five year period. Results from other commonly used administrative datasets, such as Medicaid or emergency department claims data, may show levels of congruence between true SCD genotype and ICD-9-CM genotype that vary from the results presented here. Furthermore, this study did not include individuals with SCD for whom a laboratory-confirmed genotype was not available. That is, the individuals who were neither born in California or Georgia during the years of active newborn screening nor receiving care from one of the participating clinics. Finally, there were a large number of hospitalizations during this five-year study period that contained an SCD ICD-9-CM code, but were not linked to our cohort.

These findings underscore the need for surveillance systems that can accurately identify individuals living with SCD along with their correct genotype, track their health care utilization, and monitor their quality of care by measuring adherence to practice guidelines in ways that have the potential to reduce SCD associated morbidity and mortality and improve care. Until a national registry or other data collection system is available for SCD in the US, researchers will be limited by the accuracy of ICD-9-CM or, since late 2015, ICD-10-CM codes for determining SCD genotype. Therefore, caution must be used when interpreting results and applying findings to the development of practice guidelines or recommendations for clinical care.

## Note

This project was reviewed by CDC and was determined to be a non-research, public health practice activity. Both the California Committee for Protection of Human Subjects and the Georgia Public Health Department Institutional Review Board declared the project exempt from review as a public health surveillance effort; specialty hemoglobinopathy treatment center institutional review boards similarly exempted the project from review. State data requests were reviewed by the appropriate agency, assuring data privacy safeguards were in place. The findings and conclusions in this report are those of the authors and do not necessarily represent the official position of the Centers for Disease Control and Prevention.

## Figures and Tables

**Table 1:** Description of patents with sickle cell disease and their hospital admissions, California and Georgia, 2004-2008.

	California	Georgia	Total		California	Georgia	Total
**Patients**				**Hospital Admissions**			
Number	1,093	2.868	3.961	Number	7,814	19,625	27,439
Sex				Sex			
Male	547	1,418	1,965	Male	3,750	9,087	12,837
Female	546	1,450	1,996	Female	4,064	10,538	14,602
Age group at time of last hospital admission				Age group at time of hospital admission			
0-18years	694	1,634	2,328	0-18 years	3,932	7,923	11,855
19+years	399	1,234	1,633	19+years	3,882	11,702	15,584
Sickle cell disease genotype				Sickle cell disease genotype			
Sickle cell anemia	789	2,081	2,870	Sickle cell anemia	6,189	15,744	21,933
Hb SC	223	623	E46	Hb SC	1,273	2,935	4,20E
Hb Sβ^+^ thalassemia	62	154	216	Hb Sβ^+^ thalassemia	274	878	1,152
Other	19	10	29	Other	78	68	146

**Table 2:** Positive predictive value of using Hemoglobin SS ICD-9-CM hospital discharge codes (282.61 or 282.62) to identify individuals with sickle cell anemia, California and Georgia, 2004-2008

		True SCA genotype		
Age Group	Hb SS code (282.61 or 282.62)	Yes	No	Total
0-18y	Yes	7750	556	8306
	No	2100	1334	3434
	Total	9850	1890	11740
19+y	Yes	10352	2400	12752
	No	1877	806	2683
	Total	12229	3206	15435
